# Medical College Notes

**Published:** 1890-05

**Authors:** 


### Medical College Notes.

The Fourth Annual Commencement Exercises of the Western Pennsylvania Medical College were held in the Grand Opera House, Pittsburgh, on March 27th. The degree of M. D. was conferred on twenty-nine graduates, being about twenty-five per cent. of the class in attendance during the past term. In the evening of the same day, the Alumni Association of the College—now numbering 120—was entertained by the Faculty at a banquet provided at the Seventh Avenue Hotel.

The Missouri Medical College and the St. Louis Post-Graduate School of Medicine will, in future, be under the same management. It is also announced that henceforth a three-year graded course will be required for graduation. May many more of our medical colleges follow the example of the Missouri college.

Chicago Policlinic.—Dr. N. Senn and Dr. Chr. Fenger have been elected regular Professors of Surgery in this institution. In addition to clinical work, they will present a special course in abdominal surgery twice yearly.

